# Lacticaseicin 30 and Colistin as a Promising Antibiotic Formulation against Gram-Negative β-Lactamase-Producing Strains and Colistin-Resistant Strains

**DOI:** 10.3390/antibiotics11010020

**Published:** 2021-12-24

**Authors:** Désiré Madi-Moussa, Yanath Belguesmia, Audrey Charlet, Djamel Drider, Françoise Coucheney

**Affiliations:** 1 UMR Transfrontalière BioEcoAgro 1158, Univ. Lille, INRAE, Univ. Liège, UPJV, YNCREA, Univ. Artois, Univ. Littoral Côte d’Opale, ICV—Institut Charles Viollette, F-59000 Lille, France; desire.madimoussa.etu@univ-lille.fr (D.M.-M.); yanath.belguesmia@univ-lille.fr (Y.B.); djamel.drider@univ-lille.fr (D.D.); 2 Centre Hospitalier de Lille, Centre de Biologie Pathologie, Laboratoire de Bactériologie, F-59000 Lille, France; audrey.charlet@chru-lille.fr

**Keywords:** lacticaseicin 30, colistin, Gram-negative clinical strains, beta-lactamase, synergistic activities, RT-qPCR

## Abstract

Antimicrobial resistance is a global health concern across the world and it is foreseen to swell if no actions are taken now. To help curbing this well announced crisis different strategies are announced, and these include the use of antimicrobial peptides (AMP), which are remarkable molecules known for their killing activities towards pathogenic bacteria. Bacteriocins are ribosomally synthesized AMP produced by almost all prokaryotic lineages. Bacteriocins, unlike antibiotics, offer a set of advantages in terms of cytotoxicity towards eukaryotic cells, their mode of action, cross-resistance and impact of microbiota content. Most known bacteriocins are produced by Gram-positive bacteria, and specifically by lactic acid bacteria (LAB). LAB-bacteriocins were steadily reported and characterized for their activity against genetically related Gram-positive bacteria, and seldom against Gram-negative bacteria. The aim of this study is to show that lacticaseicin 30, which is one of the bacteriocins produced by *Lacticaseibacillus paracasei* CNCM I-5369, is active against Gram-negative clinical strains (*Salmonella* *enterica* Enteritidis H10, *S. enterica* Typhimurium H97, *Enterobacter cloacae* H51, *Escherichia coli* H45, *E. coli* H51, *E. coli* H66, *Klebsiella oxytoca* H40, *K. pneumoniae* H71, *K. variicola* H77, *K. pneumoniae* H79, *K. pneumoniae* H79), whereas antibiotics failed. In addition, lacticaseicin 30 and colistin enabled synergistic interactions towards the aforementioned target Gram-negative clinical strains. Further, the combinations of lacticaseicin 30 and colistin prompted a drastic downregulation of *mcr-1* and *mcr-9* genes, which are associated with the colistin resistance phenotypes of these clinical strains. This report shows that lacticaseicin 30 is active against Gram-negative clinical strains carrying a rainbow of *mcr* genes, and the combination of these antimicrobials constitutes a promising therapeutic option that needs to be further exploited.

## 1. Introduction

Antimicrobial resistance (AMR), which has existed long before the antimicrobial era, is recognized as a serious public health threat around the world. Currently, more than 700,000 people die every year because of this phenomenon, and this number is thought to swell to 10 million by 2050, if radical actions are not taken now [1,2]. Antibiotics started to fail because of their overuse and misuse in human and animal medicine, as well as for their inappropriate recommendation. Nonetheless, unsuspected contributors such as commensal organisms, which are interconnected with microbial communities, are thought to play a role in the spread of this resistance [3]. AMR is developing rapidly to outstrip the rate at which new antimicrobials are introduced in the markets. The global effort to develop new antibiotics or modify the existing ones in order to fight AMR is considered overall to be a huge task. In spite of this worrying situation, large pharmaceutical companies have dropped out the market of antibiotics, in favor of advantageous lines of drug development such as those utilized in the cancer treatments [4,5], arguing that the cost–benefit ratio is much more favorable for other drugs.

The ways to tackle AMR and help curbing this crisis include several measures, among which the use of AMPs, which are produced by all living cells [6]. Most of AMP are of cationic nature, able to bind and interact with the negatively charged bacterial cell membranes, causing thereof a leakage of intracellular constituents, ATP depletion and cell death [7].

Bacteriocins are ribosomally synthesized antimicrobial peptides (AMPs) produced by almost all prokaryotic lineages [8,9,10], with the aim to annihilate competitors. Bacteriocins from Gram-positive bacteria are largely more abundant than their counterparts from Gram-negative bacteria and Archaea [11]. LAB-bacteriocins, which are likely the most studied ones, can be endowed with narrow spectra; therefore, they act only on a limited number of target bacteria, usually consisting of members of the same species, or endowed with broad spectra targeting other species [12]. Currently, there is not a common and universal classification scheme of bacteriocins admitted by all the scientific community. Classifications of bacteriocins are steadily reported, based on advances and progress achieved in this field [13,14,15,16]. The mode of action of LAB-bacteriocins against Gram-positives was largely studied [17]. The number of LAB-bacteriocins with activity against Gram-negatives is very low, unlike those with activity against Gram-positive bacteria. A limited number of LAB-bacteriocins active against Gram-negative bacteria was reported during the last decade [18,19,20], but their mode of action remains to be highlighted. The effectiveness of LAB-bacteriocins towards Gram-negative target bacteria can be explained by the cell-envelope structure, which consists of three layers. The cytoplasmic membrane of Gram-negative bacteria is surrounded by an outer membrane (OM), which is composed of a phospholipids bilayer, and a network of lipids and polysaccharides known as lipopolysaccharides (LPSs) [21]. The OM phospholipids are linked to the inner leaflet of the membrane, and LPS bound to the outer leaflet, which is known to cause endotoxic shock. Of note, LPS act as a barrier to many antibiotics, as well as to hydrophobic compounds [22]. Nevertheless, LPS is considered as the target of colistin, which is a polycationic antibiotic from the polymyxins groups. Colistin is known to bind to LPS and phospholipids in the OM of Gram-negative bacteria. Subsequently, it displaces divalent cations (Ca^2+^ and Mg^2+^) from the phosphate groups of membrane lipids, which leads to disruption of the OM, causing a leakage of intracellular contents and bacterial death [23]. The rapid increase in the prevalence of Gram-negative pathogens resistant to fluoroquinolones, aminoglycosides and β-lactams (carbapenems, monobactam, cephalosporins and broad-spectrum penicillins) has conducted to rehabilitation of colistin as a last valid therapeutic option [24] to treat infections caused by Gram-negative bacteria considered recalcitrant for the aforementioned drugs.

Recently, we isolated a strain of *Lacticaseibacillus paracasei* CNCM I-5369 capable to produce five distinct class II bacteriocins [20], endowed with activity against Gram-negative bacteria. This particularly interesting feature is to be exploited further as a potential therapeutic option. In 2017, the World Health Organization has categorized problematic pathogens into three categories, and those of priority level include the non-fermentative *Pseudomonas aeruginosa and Acinetobacter baumannii* as well as *Enterobacteriaceae.*

Bacteriocins produced by *L. paracasei* CNCM I-5369 were successfully produced in a heterologous host *Escherichia coli* Rosetta by Madi-Moussa [25]. Bacteriocin coded by *orf30* was particularly important because of its easy production in large quantities, unlike other ones encoded by *orf010*, *orf012*, *orf023* and *orf038* [25]. Noteworthy, bacteriocin 30, hence designed as lacticaseicin 30, was assessed in this work alone and in conjunction with colistin against a set of Gram-negative pathogenic bacteria from a clinical source, in order to design a potential therapeutic issue.

## 2. Results

### 2.1. Multifaceted Resistance of Clinical Gram-Negative Bacteria Used in This Work

The antibiogram performed on each strain used as target is given in Supplementary Materials: Table S1. The antibiotics tested here are those recommended by European Committee on Antimicrobial Susceptibility Testing (EUCAST) for *Enterobacterales* and the breakpoints used to assess their sensitivity, resistance or intermediate phenotype are from the EUCAST (https://www.sfm-microbiologie.org/, accessed on 20 November 2021). All clinical target strains tested in this work exhibited a clear resistance to colistin. Further, many of these target strains underpinned a resistance to most of penicillins tested, associated with resistance to third generation cephalosporins (Table S1). Thus, *E. coli* H45 and *Salmonella* strains showed a resistance to the penicillin but not to carbapenems and cephalosporins, indicating unmodified penicillinase activity. However, remaining *E. coli* H52 and H66 strains showed a typical AmpC beta-lactamase phenotype (Table 1) and presumably belongs to extended spectrum beta-lactamase (ESBL) group [26,27] with resistance to penicillins and at least one of the cephalosporin tested, notably cefoxitin. *E. cloacae* H51, and most of *Klebsiella* strains showed not only resistance to the aforementioned classes of antibiotics, but also to beta-lactamase inhibitors. Thus, the results using MAST disks indicate that these strains exhibited clearly an ESBL phenotype, associated with AmpC for the H77 and H51 strains, which seems to be inducible for this last one (Table 1). Of note, *K. pneumoniae* H71 showed resistance to all the antibiotics tested, including carbapenems, aminoglycosides and quinolones. Moreover, this strain exhibited an ESBL coproduced with an AmpC and a carbapenemase (Table S1 and Table 1).

### 2.2. Amplification and Sequencing of the mcr Gene Involved in the Resistance to Colistin

**Figure 1:**
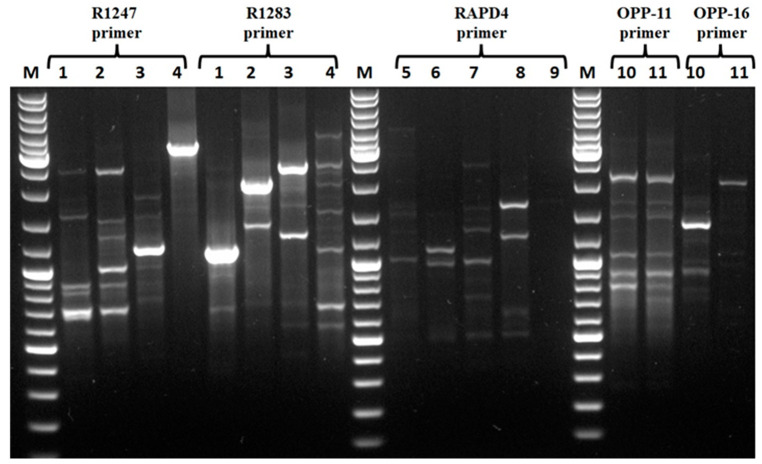
RAPD analysis of clinical Gram-negative strains, using R1247 and R1283 primers for *E. coli* strains: *E coli* 184 (1), *E coli* H45 (2), *E. coli* H51 (3), *E. coli* H66 (4), RAPD4 primer for *Klebsiella* strains: *K. oxytoca* H40 (5), *K. pneumoniae* H71 (6), *K. variicola* H77 (7), *K. pneumoniae* H79 (8), *K. pneumoniae* H79 (9), OPP-11 and OPP-16 for *Salmonella* strains: *S. enterica* Enteritidis H10 (10), *S. enterica* Typhimurium H97 (11).

Of note, ten Gram-negative bacteria isolated from diseased patients exhibiting resistance to colistin were analyzed by Random Amplification of Polymorphic DNA (RAPD) in order to discard strains with similar genetic patterns. Related to this, *E. coli* strains were obtained when primers R1247 and R1283 were used. Similar results were obtained for *Klebsiella* strains with the RAPD4 primer. When the OPP-11 primer was used with the *S. enterica* Enteritidis H10 and *S. enterica* Typhimurium H97 strains, the RAPD genetic patterns obtained were almost similar, whereas when the OPP16 primer was used the two strains exhibited clearly different patterns (Figure 1). To identify *mcr* gene associated with the colistin resistance phenotype, PCR analyses were conducted using specific primers enabling amplification of each known *mcr* gene as referred in Materiel Methods section. Of note, the *E. coli* 184 strain was used as a positive control, and this strain is known to carry a colistin resistance gene (*mcr-1*) and *E. coli* ATCC 8739 strain was used as a negative control and this strain is not harboring any *mcr* gene. Analyses of the PCR products on the agarose gel, revealed that all strains possess the *mcr-1* gene, except *E. cloacae* H51, *E. coli* 52 and *K. variicola* H77. Moreover, *E. cloacae* H51 and *K. variicola* H77 strains harbor the *mcr-9* gene, whereas the mechanism involved in the colistin resistance in *E. coli* 52 is not determined, and is not related a priori to the presence of a known *mcr* gene.

### 2.3. Expression and Characterization of the Recombinant Lacticaseicin 30

**Figure 2:**
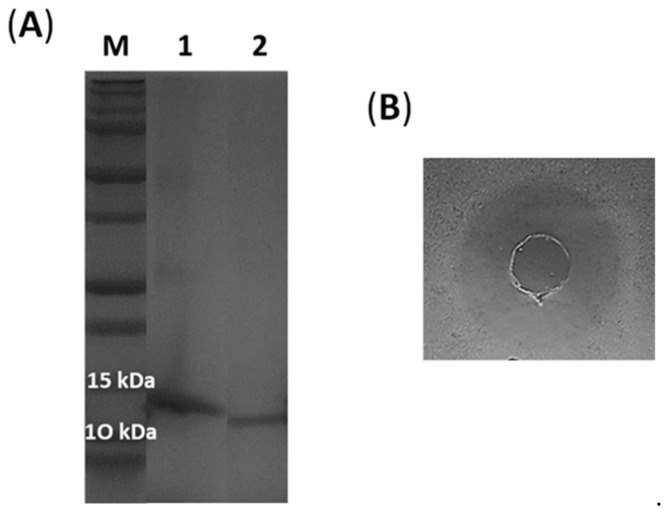
(**A**) Purified lacticaseicin 30 with (1) and without (2) histidine-tag (SDS-PAGE, 12% acrylamide), M correspond to size of proteins markers (Dual Xtra Standards, Bio-Rad). (**B**) Agar diffusion test against *E. coli* ATCC 8739 of lacticaseicin 30 (400 AU/mL) without histidine-tag.

Recombinant histidine-tagged lacticaseicin 30 was expressed in *E. coli* strain Rosetta and purified by the Ni-NTA chromatography. The histidine tag located at the N-terminal part was removed by the TEV protease. The molecular weight of the purified lacticaseicin 30 with and without tag was checked on SDS-PAGE (Figure 2A). The sizes observed on SDS-PAGE, which are 14 kDa for lacticaseicin 30 with tag, and 12 kDa for lacticaseicin 30 without tag, match approximately with those determined with the Expasy tool (https://web.expasy.org/protparam, accessed on 20 November 2021), which indicated a molecular weight of 14,088.07 Da for lacticaseicin 30 with histidine-tag and 12,339.17 Da for lacticaseicin 30 without histidine-tag. The anti-Gram-negative activity of the recombinant lacticaseicin 30 was tested at pH5 using *E. coli* ATCC 8739 as the target strain (Figure 2B), and the MIC value obtained was 40 µg/mL.

### 2.4. Lacticaseicin 30 Potentiates the Activity of Colistin and Attenuates the Expression of mcr Genes in the Clinical Strains

The combination of lacticaseicin 30 and colistin on colistin-resistant strains was investigated as indicated on Table 2. Based on FICI values obtained, a synergistic interaction was obtained between these two antimicrobials towards *E. coli* 184, *E. coli* H45, *E. cloacae* H51, *E. coli* H52, *E. coli* H66, *K. variicola* H77 and *S. enterica* Typhimurium H97 strains, as lacticaseicin 30 has contributed to significantly decrease the MIC of colistin. Nonetheless, the other strains, as indicated on Table 2, were recalcitrant as no synergistic or antagonistic effect were obtained, arguing that such synergism based on the lacticaseicin 30-colistin combination is target-strain dependent.

### 2.5. The Synergetic Interaction between Lacticaseicin 30-Colistin Downregulated Expression of mcr Gene

**Figure 3:**
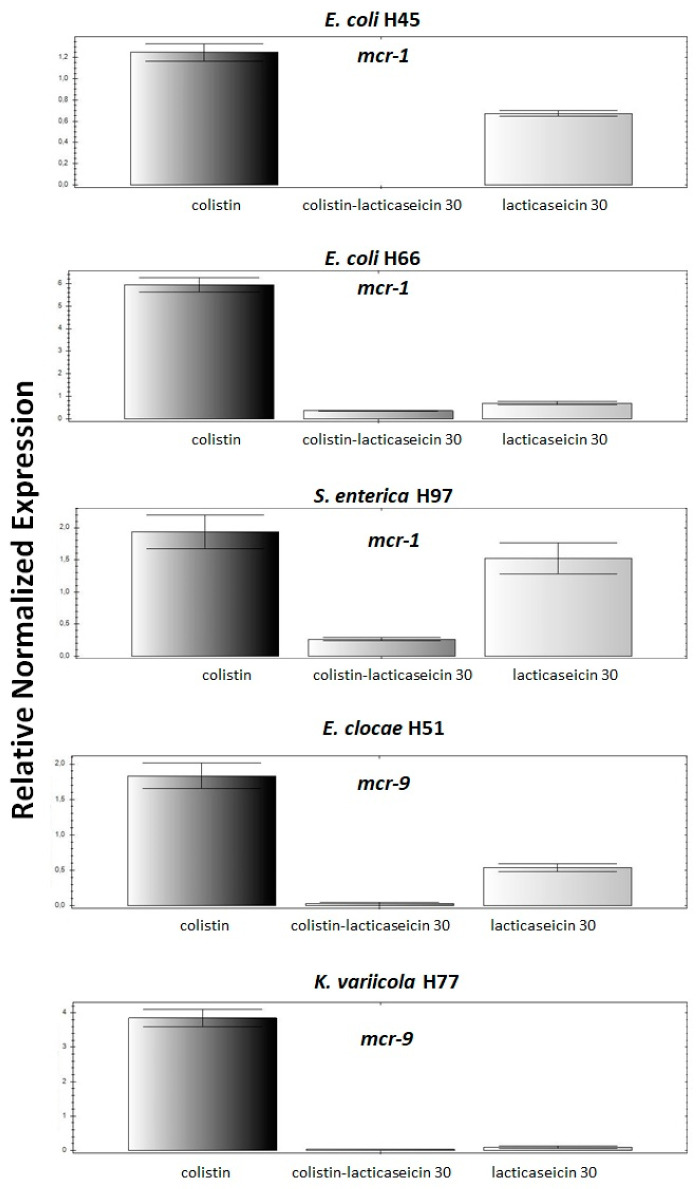
Expression of *mcr-1* or *mcr-9* gene (*mcr-1* or *mcr-9*) following bacterial treatment with colistin, lacticaseicin 30 or their combination at sub-inhibitory concentrations (MIC/2). qPCR assays performed in strains for which a synergetic interaction between lacticaseicin 30-colistin has been evidenced. Furthermore, the 16S rRNA gene was used as internal control as house-keeping gene. Three biological and technical replicates of each reaction were performed. The error bars represent a standard deviation of these replicates.

Expression of *mcr-1* gene in *E. coli* H45, *E. coli* H66 and *S. enterica* Typhimurium H97 strains and *mcr-9* gene in *E. cloacae* 51 and *K. variicola* H77 strains, for which a synergistic interaction was observed were subjected for a qPCR analysis. These strains were incubated with sub-inhibitory concentrations (MIC/2) of colistin, lacticaseicin 30 or their combination as shown on Figure 3 then the fold change in expression of the target gene (*mcr-1* or *mcr-9*) relative to the internal housekeeping gene (16S rRNA) was determined using the 2^−(ΔΔCt)^ method [28]. As expected colistin alone at a sub-inhibitory concentration induced an overexpression of the *mcr* gene in the tested strains. Remarkably, treatment of cells with lacticaseicin 30 alone at a sub-inhibitory concentration has significantly decreased the expression level of the *mcr* genes, except for *K. variicola* H77 where no expression was detected. Interestingly, we have not detected any expression of *mcr-1* genes for *E. coli* 45 following bacterial treatment with lacticaseicin 30-colistin combination at a sub-inhibitory concentration. Of note, with this combination, in the cases of *E. coli* H66 and *S. enterica* Typhimurium H97, *E. cloace* H51, *K. variicola* H77, a very low level of expression was detected.

## 3. Discussion

Aging antibiotics are currently facing ferocious resistance exerted mainly by Gram-negative bacteria, as reported in the WHO top list in 2017. This situation is considered to be a preoccupant latent crisis in the health sector worldwide [29,30]. The spreading of multiresistant pathogenic bacteria in the healthcare units impairs therapeutic options [31]. This situation is anticipated to swell and for this reason, the WHO as well as national health authorities called for global strategies to alleviate this crisis [32,33]. Colistin, which is a particular antibiotic from the polymyxins group was largely used to treat infections due to Gram-negative bacilli [34]. This antibiotic was withdrawn from the human therapeutic circuit because of its associated secondary effects such as its nephrotoxicity [35]. Nonetheless, the usage of colistin remained unmodified in the veterinary medicine, until very recently [36]. Colistin, designed also as an “old” antibiotic, is reintroduced in the therapeutic treatments as a last chance option in case of many infections associated with multiresistant and recalcitrant Gram-negative bacilli [24,34]. Resistance to colistin was usually attributed to mutations occurring on the DNA chromosome [37]. Nonetheless, in 2016, Liu et al. [38] reported the plasmid-borne transferable mobile colistin resistance (*mcr*) gene, which induces a modification of the lipid A on the lipopolysaccharides (LPS) [39]. Afterwhich, a rainbow of *mcr* genes were reported in species such as *Escherichia coli*, *Salmonella*, *Klebsiella*, *Enterobacter, Moraxella* and *Acinetobacter baumannii*. The presence of genes designated *mcr*-1 to *mcr*-10 represent the risk of exacerbating this crisis [40,41]. Within the panel of innovative strategies expected to tackle AMR, the usage of AMP such as bacteriocins offers a novel hope [42,43].

In light of this, we establish in this work that lacticaseicin 30, a novel class II bacteriocin is active against a set of clinical Gram-negative pathogens, and potentiates the activity of colistin on strain carrying *mcr*-1 and *mcr*-9 genes, including multiresistant *E. coli*, *Salmonella*, *Klebsiella* and *Enterobacter*. Remarkably, clinical Gram-negative strains displaying sensitivity to lacticaseicin 30 are also characterized by the presence of an AmpC phenotype, due to a mutation of the beta-lactamase promoter [27]. Moreover, some of these strains exhibited ESBLs. This heterogeneous family of bacterial enzymes, discovered during the 1980s in Europe [44], are encoded by genes located on plasmids, or could result from a mutation of a natural gene, thus leading to a synthesis of TEM- and SHV- modified enzymes [45,46]. Mutations responsible for ESBLs broaden the spectrum of these enzymes and, therefore, allow the hydrolysis of a wide variety of antibiotics (penicillin monobactams and cephalosporins of third generation such as ceftazidime and cefotaxime [47,48]. This resistance was reported as being frequently associated with the resistance to fluoroquinolones [47]. The increased rate of infections associated with ESBL-bearing bacteria constitutes a therapeutic challenge, as only cephalosporins or quinolones are recommended [46,48]. In the present study, we report synergistic interactions between lacticaseicin 30 and colistin, and clinical strains evaluated as resistant to colistin have seen their breakpoints decreased, thus becoming sensitive to such antibiotics, except for *K. pneumoniae*, *K. oxytoca* and *S. enterica* Enteritidis ones. The mode of action of both antimicrobials remains to be elucidated, although a mode of action organized in two steps could be claimed. In a former study [20], we reported that bacteriocins produced by *L. paracasei* CNCM I-5369 are not explicitly targeting the LPS of Gram-negatives, but colistin uses LPS to undergo its activity. Therefore, lacticaseicin 30 could take advantage from a potential breach caused by colistin, to proceed with its own activity on the cell-membrane or inside the bacterial cell by cooperative mode of action. Indeed, colistin have a well-known mode of action, disturbing the outer cell membrane of by displacing Ca^2+^ and Mg^2+^ divalent cations from the phosphate groups of membrane lipids, leading to leakage of intracellular contents and bacterial death [24,49], whereas bacteriocins act generally by pore-forming mode of action provoking permeabilization of the target bacteria cell membrane [8,49]. Synergistic interaction between antimicrobials was reported as an important mean to decrease the number of drugs used, to elude the bacterial resistance and to control any undesirable secondary effect of drugs and finally provide an efficient and affordable therapeutic solution [50]. Related to that, bacteriocins were shown to potentiate a wide range of molecules including nanoparticles [43]. It should be noted that different combinations of bacteriocins and antibiotics, including colistin were already reported in the literature [49,51,52,53,54]. Most of these studied formulations involved the use of nisin as partner of colistin.

Nisin, a class I bacteriocin, is categorized as food additive E234 in the EU under Annex II of Regulation (EC) 1333/2008. This “old” bacteriocin was tested as well with other antibiotics such as tetracycline, methicillin and vancomycin, and the resulting combinations enabled synergistic interactions against multiresistant Gram-positive and Gram-negative pathogenic bacteria [55,56,57,58]. Further bacteriocins, such as leaderless enterocin 14 (Ent DD14) from *Enterococcus faecalis* 14, were shown to potentiate other antibiotics such as methicillin [59] and erythromycin [51] without causing damages on mice microbiota or impairing their main organs such as spleen, liver and colon [60]. The present study provides for the first time an insightful information on the relevancy of alternative strategy combining the lacticaseicin 30 with the colistin, on multiresistant clinical strains harboring the *mcr* gene. Indeed, lacticaseicin 30 or lacticaseicin 30+ colistin enabled a clear downregulation of *mcr*-1 or *mcr*-9 expression transcript. Similar effects were observed by Zgheib et al. [61] and Belguesmia et al. [59] when assessing the impact of EntDD14 on the expression of genes coding for virulence factors in *Clostridium perfringens* and MRSA-SA1 strains. From the twelve strains considered in this study, ten possess the plasmidic *mcr-1* gene, sharing common regulation features [62]. Of note, the expression of the *mcr-1* gene appears to be closely linked to phosphoesterase encoding gene pap2, present downstream of *mcr-1*, forming an *mcr-1-pap2* cassettes in plasmid. The remaining two strains, *E. cloacae* H51 *K. variicola* H77, harbor the *mcr-9* variant which was initially isolated from *Salmonella enterica* Serotype Typhimurium Isolate [63]. Interestingly, as for *mcr-1* gene [64], this new variant was found to be inducible in the presence of sub-inhibitory concentration of colistin [65]. The encoded MCR-9 share 84% of identity with MCR-BG encoded by chromosomic gene harbored by *Buttiauxella gaviniae*, whereas the *mcr-1*, originated from *Moraxella* species, share about 36% of identity [66]. Notably, a putative two-component system corresponding to a histidine kinase sensor (QseC), and its cognate partner (QseB) were found downstream to the *mcr-9* gene and possibly implied in the regulation of this gene. Despite these differences, the combination of lacticaseicin 30 and colistin enabled to downregulate the expression level of both *mcr* genes found in the clinical strains studied. The exact mechanisms by which the bacteriocins could impact the expression level of such genes, coding for antibiotic resistance or virulence factors, remain to be elucidated. The interaction of bacteriocins with promoter region of these genes or disruption of transcription machinery in the target cells could be one of the possible mechanisms implied, which will be the subject of further investigations in direct line with the perspective of this research work.

## 4. Materials and Methods

### 4.1. Bacterial Strains and Culture Conditions

All target strains used in this study and their characteristics are reported in Table 3. These strains were grown in brain heart infusion broth (BHI, Sigma-Aldrich Saint-Louis, MO, USA) at 37 °C without shaking. All pathogenic Gram-negative bacteria were obtained from diseased patients at Lille University Hospital (France), except for *Escherichia coli* ATCC 8739 and *E. coli* 184 used as controls. *E. coli* Rosetta -T7-6his-30 strain (Table 3) was used to produce recombinant lacticaseicin 30. This strain was grown in Luria-Bertani broth (LB) at 37 °C with shaking at 160 rpm [25].

### 4.2. Antibiotic Susceptibility of Clinical Strains

The antibiogram of each target strain was performed with the VITEK^®^ 2 system (Biomerieux, Marcy-l’Étoile, France) using the routinely recommended protocol. The UMIC microplate method (Biocentric, Bandol, France) was used to determine the sensibility of these clinical strains for colistin [67]. For the determination of the ESβL and AmpC production, we used the MASTDISCS^®^ Combi (Mast Group LTD, Bootle, UK), according to the manufacturer indication.

### 4.3. Genomic DNA Extraction

Genomic DNA (gDNA) was extracted from 1 mL of each strain suspension using the “NucleoSpin Microbial DNA” kit from Macherey-Nagel (Düren, Germany), and then was checked on agarose gel electrophoresis [68], and quantified with the NanoDrop Spectrophotometer (Thermo Fisher Scientific, Waltham, MA, USA).

### 4.4. Detection of mcr Gene in the Clinical Strains Exhibiting a Colistin Resistant Phenotype

The identification of the type of *mcr* gene, involved in the resistance for colistin resistance, was performed by the polymerase chain reaction (PCR) method, using the appropriate oligonucleotide primers listed in Table 4. Each PCR reaction was realized in a final volume of 25 µL, containing 12.5 µL of DreamTaq PCR Master Mix (2X) (Thermo Fisher Scientific), 1.25 µL of each appropriate oligonucleotide primer (10 µM), 8 µL of nuclease free water and 2 µL of gDNA. The PCR program consisted in the following steps: 1 cycle of denaturation at 94 °C for 15 min, followed by 30 cycles of denaturation at 94 °C for 30 s, annealing at melting temperature (Tm, Table 4) for 30 s and elongation at 72 °C for 60 s, and a final cycle of elongation at 72 °C for 10 min.

### 4.5. Random Amplification of Polymorphic DNA PCR (RAPD PCR)

The R1247 (AAGAGCCCGT) and R1283 (GCGATCCCCA) oligonucleotide primers (Table 4) were used to establish the genetic profiles of *E. coli* strains, [71]. The RAPD4 (AAGACGCCGT) oligonucleotide primer (Table 4) was used to identify and select distinct *Klebsiella* strains [72], and the OPP-16 (CCAAGCTGCC) and OPP-11 (AGTCGGGTGG) oligonucleotide primers (Table 4) permitted to identify and select the distinct *Salmonella* strains [73] and discard redundant ones. The RAPD-PCR reaction was performed in a final volume of 25 μL containing 12.5 µL of DreamTaq PCR Master Mix (2X), 2 μL of appropriate primer (10 µM), 8.5 μL of nuclease-free water, and 2 μL of gDNA. The PCR program started with a first step of 10 min at 95 °C, followed by 40 cycles of denaturation at 95 °C for 1 min, annealing at 38/36 °C for 1 min and elongation at 72 °C for 2 min. Finally, a second extension step of 10 min at 72 °C was performed.

### 4.6. Production and Purification of Recombinant Lacticaseicin 30

To produce a recombinant lacticaseicin 30, a preculture of *E. coli* Rosetta-T7-6his-030, harboring the plasmid carrying the gene coding for lacticaseicin 30 (*orf030*) was used to inoculate 1% (*v*/*v*) of LB broth supplemented with 100 µg/mL of ampicillin (Sigma-Aldrich). Expression of lacticaseicin 30 was induced by the addition of 0.5 mM isopropyl β-d-1-thiogalactopyranosid (IPTG, Sigma-Aldrich) and cells were incubated for three additional hours at 37 °C with shaking at 160 rpm. Cells were collected by centrifugation (10 min, 11,000× *g* at 4 °C), and re-suspended in Tris−HCl buffer containing 20 mM Tris–HCl pH 8 and 300 mM NaCl. To recover the soluble recombinant bacteriocin fraction from the cytoplasm, cells were lysed by sonication three times during 40 s at 180 Watt (OmniRuptor 4000 Ultrasonic Homogenizer, Omni International, Georgia, GA, USA). After their lysis, the bacteriocin was purified by the Ni-NTA chromatography as recently reported by Madi-Moussa et al. [25]. The histidine tag was removed with Tev protease (Sigma-Aldrich) following the recommended instructions. The purity of the bacteriocin was checked on Tricine-SDS-PAGE [74]. After purification, the final concentration of lacticaseicin 30 was determined using the bicinchoninic acid assay protein kit (BCA, Sigma-Aldrich).

### 4.7. Determination of Minimal Inhibitory Concentrations (MICs) in Checkerboard Assays

The antimicrobial activity of lacticaseicin 30 was determined using the agar diffusion test method [75]. Interaction between this bacteriocin and colistin were determined with the checkerboard MIC method as previously described by Ahmad et al. [76]. MICs are defined as the lowest concentration of an antimicrobial agent that inhibits the visible growth of a microorganism after an overnight incubation at 37 °C [77]. Given that lacticaseicin 30 is only active at pH 5, BHI broth, colistin sulfate salt (Sigma-Aldrich) and lacticaseicin 30 were acidified to pH 5 with acetic acid (Sigma-Aldrich). Similarly, BHI broth alone (negative control) was also adjusted to pH 5. The checkerboard assay of lacticaseicin 30 and colistin were performed using seven doubling dilutions for each combined component [36]. After that, microplates were inoculated with ~10^6^ colony forming units/mL of the target strain, in a final volume of 200 µL per well, and incubated overnight at 37 °C without agitation. The fractional inhibitory concentration index (FICI) was calculated for each combination using the following formula: FICA + FICB = FICI, where FICA = MIC of drug A in combination/MIC of drug A alone, and FICB = MIC of drug B in combination/MIC of drug B alone. The FICI was interpreted as follows: synergy = FICI ≤ 0.5; indifference = FICI > 0.5 ≤ 4; antagonism = FICI > 4 [78].

### 4.8. Total RNAs Extraction and cDNA Synthesis

Total RNA was extracted from a 5 mL culture of the target strain treated for 24 h with colistin or lacticaseicin 30 or both of them at subinhibitory concentrations (MIC/2) using the “NucleoSpin RNA” kit (Macherey-Nagel) and following the recommendations of the manufacturer. After controlling the RNA integrity on agarose gel following an electrophoresis performed at a constant voltage of 120 V for 45 min, 1 µg of RNA from each sample was first treated with DNase (Thermo Fisher Scientific) and converted into cDNA using the Revert Aid H Minus First Strand cDNA Synthesis Kit (Thermo Fisher Scientific), following the recommendations of the manufacturer.

### 4.9. Quantitative PCR (qPCR) and Analysis of mcr Genes Expression

Each qPCR reaction containing 12.5 µL of Takyon™ No ROX SYBR 2X MasterMix blue dTTP (Eurogentech, Seraing, Belgium), 1.25 µL of each appropriate primer (Table 4), 2 µL of cDNA and 8 µL of nuclease free water, was performed in triplicate using CFX Connect Real-Time PCR Detection System thermocycler (Bio-Rad, Hercules, CA, USA). The qPCR program consists of an initial step at 95 ° C for 10 min followed by 45 cycles of 95 °C for 15 s, 58 °C for 1 min, and 72 °C for 30 s and an additional step starting between 90 °C and 58 °C was performed to establish a melting curve and verify the specificity of amplicon product [79]. Then, the threshold cycle (Ct) values for each qPCR reaction were obtained using Bio-Rad’s CFX Manager software. The Ct value is the basis for the calculation of the relative quantification, corresponding to the expression of the target gene (*mcr* gene) compared with the house-keeping gene (*16S rRNA* gene), as demonstrated in [28]. The analysis of the relative expression of the target genes was determined using the 2^−(ΔΔCt)^ method as previously described by [25,80].

## 5. Conclusions

Bacteriocins are produced by a large number of bacterial species including those from the human microbiota [11]. Their immediate application in the human and veterinary health must be considered as a priority. These molecules can be part of the solution for the announced AMR crisis. Bacteriocins can be explored in different ways. First, by favoring their production in situ [11], or as potentiating agent of aging antibiotics as reported in a number of clinical studies. The present study enlarged the portal of bacteriocins as molecules of medical interest and permitted to claim that LAB-bacteriocins can act alone or in combination with colistin against multiresistant Gram-negative bacteria. The in vivo assessment of lacticaseicin 30 alone or in combination with colistin constitutes our next goal.

## Figures and Tables

**Table 1:** Beta-lactamase phenotype.

Strains	D72A	D72B	D72C	D72D	D72E	Conclusion
*Enterobacter cloacae* H51	R	S	R	S	R	EsβL + AmpC Inducible
*E. coli* H52	R	R	S	S	I	AmpC
*E. coli* H66	R	R	S	S	S	AmpC
*Klebsiella oxytoca* H40	R	S	R	S	S	EsβL
*K. pneumoniae* H71	R	R	R	R	R	EsβL + AmpC + Carbapenemase
*K. variicola* H77	R	S	I	S	S	EsβL + AmpC
*K. pneumoniae* H79	R	S	R	S	S	EsβL

Legend. D72A: Cefpodoxime 10 μg discs; D72B: Cefpodoxime 10 μg + EsβL inhibitor discs; D72C: Cefpodoxime 10 μg + AmpC inhibitor discs; D72D: Cefpodoxime 10 μg + EsβL inhibitor + AmpC inhibitor discs; D72E: Cefpodoxime 10 μg + EsβL inhibitor + AmpC inducer discs; R: Resistant; I: Intermediary; S: sensitive.

**Table 2:** Effects of the lacticaseicin 30-colistin combination against Gram-negative target bacteria displaying resistance to colistin.

Strains	*mcr* Gene	Lacticaseicin 30 (µg/mL)	Colistin (µg/mL)	FIC Lacticaseicin 30	FIC Colistin	FIC I	Conclusions
*E. coli* 184	*mcr-1*	40	8	0.25	0.125	0.375	Synergism
*Enterobacter cloacae* H51	*mcr-9*	40	16	0.25	0.25	0.5	Synergism
*E. coli* H45	*mcr-1*	40	8	0.25	0.25	0.5	Synergism
*E. coli* H52	-	40	16	0.125	0.25	0.375	Synergism
*E. coli* H66	*mcr-1*	40	16	0.125	0.25	0.375	Synergism
*Klebsiella oxytoca* H40	*mcr-1*	40	64	1	1	2	Indifference
*K. pneumoniae* H71	*mcr-1*	40	8	0.5	1	1.5	Indifference
*K. variicola* H77	*mcr-9*	40	8	0.25	0.25	0.5	Synergism
*K. pneumoniae* H79	*mcr-1*	40	4	0.25	0.5	0.75	Indifference
*K. pneumoniae* H87	*mcr-1*	40	32	1	1µ	2	Indifference
*Salmonella enterica* H10	*mcr-1*	40	4	1	1	2	Indifference
*S. enterica* Typhimurium H97	*mcr-1*	40	8	0.125	0.125	0.25	Synergism

-: unknow.

**Table 3:** Bacterial strains used in this work.

Strains	Relevant Characteristics
Producing strain	
*Escherichia coli* Rosetta-T7-6His-030	Strain harboring the plasmid carrying the *orf030* gene, encoding the lacticaseicin 30 [25]
Control target strains	
*Escherichia coli* ATCC8739	[20]
*Escherichia coli* 184	Strain harboring the plasmid carrying the *mcr-1* gene, responsible for colistin resistance [20]
Gram-negative clinical target strains	
*Enterobacter cloacae* H51	This work
*Escherichia coli* H45	This work
*Escherichia coli* H52	This work
*Escherichia coli* H66	This work
*Klebsiella oxytoca* H40	This work
*Klebsiella pneumoniae* β-lactamase H71	This work
*Klebsiella variicola* β-lactamase H77	This work
*Klebisella pneumoniae* β-lactamase H79	This work
*Klebsiella pneumoniae* β-lactamase H87	This work
*Salmonella enterica* Enteritidis H10	This work
*Salmonella enterica* Typhimurium H97	This work

**Table 4:** Oligonucleotide primers used in this study and their target gene.

Target Gene	Name	Sequence 5′ → 3′	Tm (°C)	Size (bp)	References
**Primers used for *mcr* gene detection**					
***mcr-1***	F-*mcr*-1	AGTCCGTTTGTTCTTGTGGC	58	320	[69]
	R-*mcr*-1	AGATCCTTGGTCTCGGCTTG			
***mcr-2***	F-*mcr*-2	CAAGTGTGTTGGTCGCAGTT	58	715	[69]
	R-*mcr*-2	TCTAGCCCGACAAGCATACC			
***mcr-3***	F-*mcr*-3	AAATAAAAATTGTTCCGCTTATG	58	929	[69]
	R-mcr-3	AATGGAGATCCCCGTTTTT			
***mcr-4***	F-*mcr*-4	TCACTTTCATCACTGCGTTG	58	1116	[69]
	R-*mcr*-4	TTGGTCCATGACTACCAATG			
***mcr-5***	F-*mcr*-5	ATGCGGTTGTCTGCATTTATC	58	1644	[69]
	R-*mcr*-5	TCATTGTGGTTGTCCTTTTCTG			
***mcr-6***	F-*mcr*-6	AGCTATGTCAATCCCGTGAT	52	252	[70]
	R-*mcr*-6	ATTGGCTAGGTTGTCAATC			
***mcr-7***	F-*mcr*-7	GCCCTTCTTTTCGTTGTT	50	551	[70]
	R-*mcr*-7	GGTTGGTCTCTTTCTCGT			
***mcr-8***	F-*mcr*-8	TCAACAATTCTACAAAGCGTG	53	856	[70]
	R-*mcr*-8	AATGCTGCGCGAATGAAG			
***mcr-9***	F-*mcr*-9	TTCCCTTTGTTCTGGTTG	55	1011	[70]
	R-*mcr*-9	GCAGGTAATAAGTCGGTC			
***mcr-10***	F-*mcr*-10	GGACCGACCTATTACCAGCG	64		[41]
	R-*mcr*-10	GGCATTATGCTGCAGACACG			
**Primers used for qPCR analyses**					
***mcr-1***	F-*mcr-1*-qPCR	CGCGATGCTACTGATCACCA	58	100	In this study
	R-*mcr*-1-qPCR	AAAATAACTGGTCACCGCGC			
***mcr-9***	F-*mcr*-9-qPCR	ATCCGTTCCGTGCATGTTCT	58	100	In this study
	R-*mcr*-9-qPCR	CACCGGTTTTCTGCACGATG			
**16sRNA**	*F-16sRNA-qPCR*	GTAGGTGGCAAGCGTTATCC	58	101	In this study
	R-16sRNA-qPCR	GATGCGCTTCCTCGGTTAAG			

## Supplementary Materials

The following supporting information can be downloaded at: https://www.mdpi.com/article/10.3390/antibiotics11010020/s1, Table S1: Antibiograms of clinical Gram-negative bacteria used in this work.
