# Supplementary material for: Lacticaseicin 30 and Colistin as a Promising Antibiotic Formulation against Gram-Negative β-Lactamase-Producing Strains and Colistin-Resistant Strains
Source: Antibiotics (Basel). 2021 Dec 24;11(1):20. doi: 10.3390/antibiotics11010020 (PMC8772908; doi:10.3390/antibiotics11010020)
Supplement: Supplementary file 1 [file antibiotics-11-00020-s001.zip › antibiotics-1503592-supplementary Table S1.pdf]

Supplementary Materials

Table S1. Antibiograms of clinical Gram-negative bacteria used in this work.

	E. cloacae **H51**	*E. coli* **H45**	*E. coli* **H52**	*E. coli* **H66**	*K. oxytoca* **H40**	*K. pneumoniae* **H71**	*K. variicola* **H77**	*K. pneumoniae* **H79**	*K. pneumoniae* **H87**	*S. enterica* **Enteritidis H10**	*S. enterica* **Typhimurium H97**
**Penicillins**											
Ampicilin	R	R	R	R	R	R	R	R	R	S	R
Amoxicillin/ clavulanic acid	R	I	R	R	S	R	S	R	R	S	R
Ticarcillin	R	R	R	R	R	R	R	R	R	S	R
Ticarcillin/ clavulanic acid	R	I	R	R	R	R	I	R	R	S	R
Temocillin	-	S	S	-	-	-	-	R	-	-	-
Piperacillin	R	R	R	R	R	R	R	R	R	S	R
Piperacillin/ tazobactam	-	S	I	R	R	R	S	R	R	S	S
Imipenem	S	S	S	S	S	R	S	S	S	S	S
Ertapenem	S	S	S	S	S	R	S	S	S	S	-
**Cephalosporins**											
Cefoxitin	R	S	R	R	S	R	S	S	R	S	S
Cefotaxime	R	S	S	S	R	R	R	R	R	S	S
Ceftriaxone	S	S	S	S	R	R	R	R	R	S	S
Ceftazidime	R	S	I	S	R	R	S	R	R	S	S
Cefepim	R	S	S	S	S	R	S	R	R	S	S
**Oral cephalosporins**											
Cefixime	R	S	R	R	R	R	R	R	R	S	S
**Aminoglycosides**											
Tobramycin	-	S	S	S	R	R	R	R	R	-	-
Amikacin	-	S	S	S	S	-	I	I	I	-	S
Gentamycin	R	S	S	S	R	R	R	R	R	-	R
**Tetracyclines**											
Tigecycline	-	S	S	S	R	-	S	I	R	S	-
**Polypeptides**											
**Colistin**	**R**	**R**	**R**	**R**	**R**	**R**	**R**	**R**	**R**	**R**	**R**
**Sulfonamide and combination**											
Trimethoprim	R	R	S	S	R	-	R	R	R	S	
Trimethoprim/ sulfamethoxazole	R	R	S	S	R	-	R	R	S	S	R
**Nitrofurans**											
Nitrofuran	-	S	S	S	R	-	-	R	-	R	S
**Quinolones**											
Nalidixic acid	R	-	S	S	R	R	R	R	-	R	S
Ofloxacin	-	-	S	S	S	R	R	R	-	R	S
Levofloxacin	-	-	S	S	S	R	R	R	-	R	-
Ciprofloxacin	-	-	S	S	S	R	R	R	-	R	S
**Diverses**											
Fosfomycin	-	S	-	-	-	-	-	-	-	-	S

Legend. R : Resistant; I : Intermediary; S: sensitive (-): not determined.
